# Carbolic Acid

**Published:** 1863-12

**Authors:** 


Carbolic Acid.—This peculiar substance seems to have almost as many names as the wandering Jew, and, in defectively compiled chemical works, each name is set down as meaning a different substance. It received the above from its discoverer, Runge, in 1834, and it is now also called phenic acid, phenol, phenic alcohol, and the hydrate of phenyle. It may be obtained synthetically as well as analytically. The distinguished chemist Bertholet has made it by passing alcohol and acetic vapors through a porcelain tube heated to redness. It is, however, commonly obtained from the oil of coal-tar. When this oil is submitted to fractional distillation, the part which passes over between 160° and 190° of temperature is treated with hot saturated caustic and some powdered potash, and a mass of crystals is obtained which is separated by decantation. When these are dissolved in water the solution separates into layers; the one light and oily, the other heavy and watery. The latter is separated and treated with hydrochloric acid, which sets free carbolic acid. To make it perfectly pure, however, it is digested with fused chloride of calcium and redistilled twice; when, upon cooling slowly, it forms into a solid, colorless mass [C2 H6 O H O]. It has an odor resembling creosote, and is sometimes sold for it; but the latter is a distinct substance. Carbolic acid burns with a reddish flame, and boils at 180°. It is slightly soluble in water but very soluble in alcohol, ether, acetic acid, glycerin, and some volatile oils. It acts very energetically upon the skin; a weak aqueous solution coagulates albumen, and acts as a strong antiseptic. Putrid meet, fish, and fermenting animal substances instantly lose their disgusting odor when treated with a solution of carbolic acid. It is employed, mixed with plaster-of-Paris, in disinfecting powders, as it arrests fermentation and destroys infection. Weak solutions of it instantly destroy the lowest forms of animal and vegetable life; and ink, solutions of glue, and the juices of vegetables are prevented from becoming moldy by an addition of a very small quantity of it. A strong solution of it kills the eggs of ants, caterpillars, and the larvæ of flies. When applied lightly to the human skin, the latter smarts for about an hour, the epidermis becomes wrinkled, and remains red and somewhat inflamed for about twenty days. When a very minute quantity of it is taken into the stomach it seems to act upon the nerves, producing insensibility almost instantaneously.

DEATHS by chloroform are reported almost weekly in the London medical journals. Six have occurred during the last two or three months. But this fearful mortality does not seem to attract unusual attention, nor does it lead to any very practical efforts to correct the evils attendant upon its administration. A Chloroform Committee has been appointed to report upon the employment of this agent, but no other step has been taken. As usual, these cases have occurred where a very slight operation was about to be performed. It is probable that the fault generally lies in the manner of administration. This duty is generally intrusted to the junior physician present, and either to his ignorance or carelessness the fatal issue of the case may be traced. It would be better to discard this agent altogether than intrust it to incompetent hands.—*Am. Med. Times, Dec.* 12.

